# A Rare Case of Acute Idiopathic Gastric Necrosis

**DOI:** 10.7759/cureus.94510

**Published:** 2025-10-13

**Authors:** Deepam Yadav, Krishnanand Anand, Sachin Goel

**Affiliations:** 1 General Surgery, LN Medical College and Research Center, Bhopal, IND

**Keywords:** gastric dilatation, gastric ischemia, idiopathic gastric necrosis, intestinal obstruction, stomach

## Abstract

Acute idiopathic gastric necrosis is a rare, life-threatening condition resulting from sudden gastric ischemia and subsequent wall necrosis. Here, we describe a 14-year-old boy who presented with upper intestinal obstruction, vomiting, abdominal distension, severe pain, and failure to pass flatus or stool. Imaging revealed gastric and duodenal dilatation, indicating proximal obstruction. Exploratory laparotomy identified a gangrenous patch on the anterior gastric wall, necessitating resection and gastrojejunostomy. Despite postoperative complications, including gastric perforations and evisceration, the patient recovered following timely re-intervention. This case underscores the need for early diagnosis, prompt surgical management, and multidisciplinary care to prevent further complications and reduce mortality.

## Introduction

Acute idiopathic gastric necrosis represents a challenging and potentially life-threatening condition encountered in emergency surgery, characterized by sudden-onset gastric ischemia leading to tissue death without an identifiable underlying cause [1,2]. The condition demonstrates a predilection for older adult populations, making its occurrence in pediatric and adolescent age groups particularly unusual and clinically significant [3]. This rare pathological entity poses significant diagnostic and therapeutic challenges due to its rapid progression and nonspecific clinical presentation [4]. The underlying pathophysiology involves compromised gastric vascular supply resulting in ischemic necrosis of gastric tissue [1]. Distinguished from secondary gastric necrosis with identifiable etiology, such as mesenteric ischemia or caustic substance ingestion, the idiopathic form develops without apparent precipitating factors [5], necessitating heightened clinical vigilance for optimal patient outcomes [6]. Clinical manifestations typically encompass severe abdominal pain, persistent emesis, abdominal distension, and features suggestive of upper gastrointestinal obstruction [1,3]. The nonspecific symptomatology frequently contributes to diagnostic uncertainty and potential delays in definitive management [4]. This condition carries substantial risks of morbidity and mortality, underscoring the critical importance of expeditious recognition and appropriate surgical intervention [5].

Radiological evaluation serves as a cornerstone in identifying gastric dilatation and associated pathological changes, though definitive diagnosis frequently necessitates direct surgical assessment [7]. Therapeutic management conventionally involves excision of necrotic gastric tissue with subsequent reconstruction, commonly utilizing gastrojejunostomy techniques [4,8]. The postoperative period may be complicated by anastomotic complications, perforation, and septic sequelae, requiring intensive monitoring and potentially necessitating additional surgical procedures [5]. The infrequent occurrence of this condition, especially within younger patient populations, results in limited collective clinical experience, rendering individual case documentation valuable for advancing therapeutic understanding and management protocols [3,4]. Ingestion of caustic substances is a common toxicological emergency, often resulting in significant morbidity and mortality. Accidental ingestion of these toxic agents is most prevalent among children, who may encounter household cleaners and other hazardous substances out of curiosity. In contrast, adults often ingest caustic agents in the context of self-harm or suicide attempts. These agents can cause extensive damage to the gastrointestinal tract, leading to serious complications, including perforation, strictures, and systemic toxicity [9]. Previous reports have documented various presentations, including cases secondary to volvulus and those requiring total gastrectomy with complex reconstructive procedures [10,11].

We present the management of acute idiopathic gastric necrosis in a 14-year-old male patient, emphasizing the distinctive challenges encountered in pediatric cases and demonstrating the significance of coordinated multidisciplinary care in achieving positive outcomes despite procedural complications. This clinical experience adds to the existing knowledge base regarding pediatric gastric necrosis and reinforces the fundamental principles of clinical recognition, timely intervention, and comprehensive perioperative management in addressing this complex surgical condition. Our results indicate that through skilled surgical technique and intensive supportive care, challenging cases of acute idiopathic gastric necrosis can achieve successful outcomes.

## Case presentation

**Figure 1:**
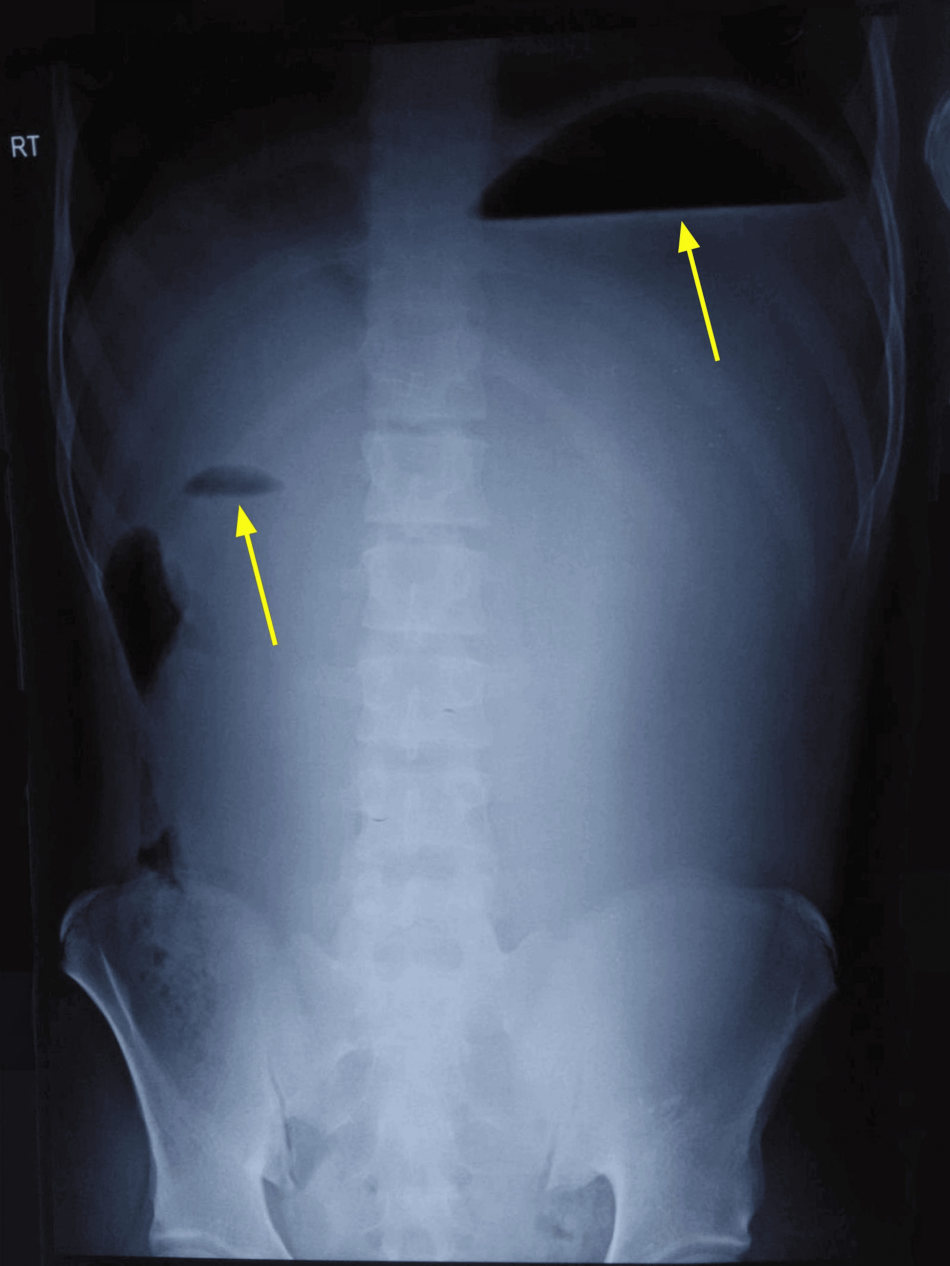
Abdominal X-ray revealing a potential “double bubble” sign suggestive of duodenal obstruction (yellow arrows)

A 14-year-old boy presented to the emergency department with a prior diagnosis of upper intestinal obstruction with gastric necrosis. This diagnosis followed an exploratory laparotomy performed at a private hospital for suspected intestinal obstruction. The patient reported with three-day history of repeated vomiting, abdominal distension, severe abdominal pain, and an inability to pass flatus and motion. Preoperative imaging at the initial hospital included an erect abdominal X-ray (Figure 1), which revealed a possible "double bubble" sign indicative of duodenal obstruction. 

**Figure 2:**
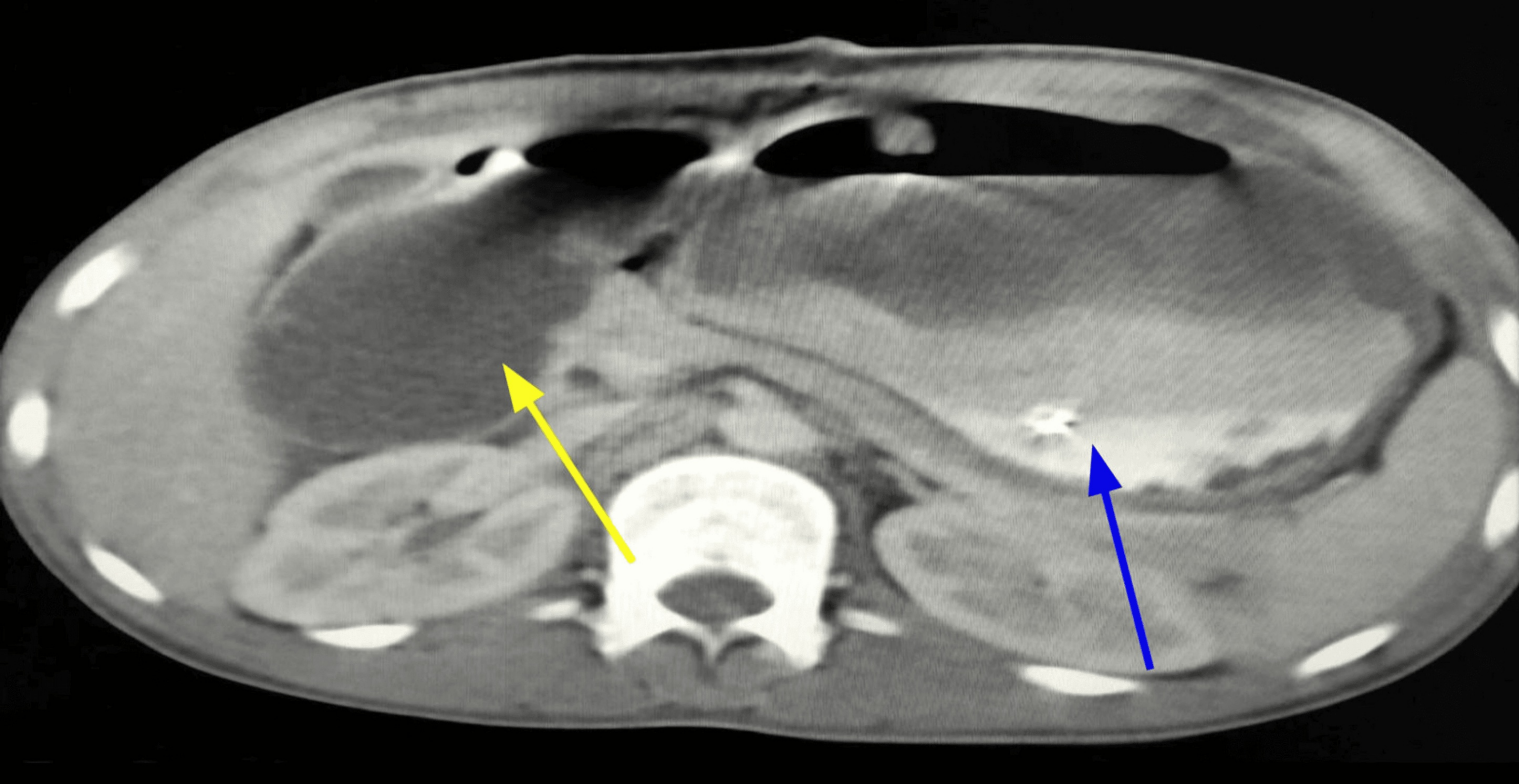
A contrast-enhanced computed tomography (CECT) of the abdomen showing a dilated stomach (blue arrow) and a dilated third segment of the duodenum (yellow arrow)

Ultrasound of the abdomen showed a grossly dilated stomach occupying the entire abdominal cavity, consistent with proximal duodenal obstruction. During the initial laparotomy, marked dilatation of the stomach and duodenum up to the second portion was noted, along with a gangrenous patch involving the majority of the anterior gastric wall. No intraperitoneal contamination or peritoneal pathology was identified, and the abdomen was closed without further intervention. The patient was subsequently referred to our center for definitive management. On admission, the patient was hemodynamically unstable, with a pulse rate of 120 bpm, respiratory rate of 28 bpm, blood pressure of 90/50 mmHg, and oxygen saturation of 92%. Laboratory evaluation revealed lactic acidosis and significant electrolyte derangements. The patient was admitted to the intensive care unit (ICU) for immediate resuscitation. Double contrast-enhanced computed tomography (CECT) of the abdomen (Figure 2) showed significant gastric dilatation with food residue, dilatation of the duodenum with a transition zone at the D3 level, mild dilatation of the lower esophagus, left-sided pleural effusion, ascites, and pneumoperitoneum.

**Figure 3:**
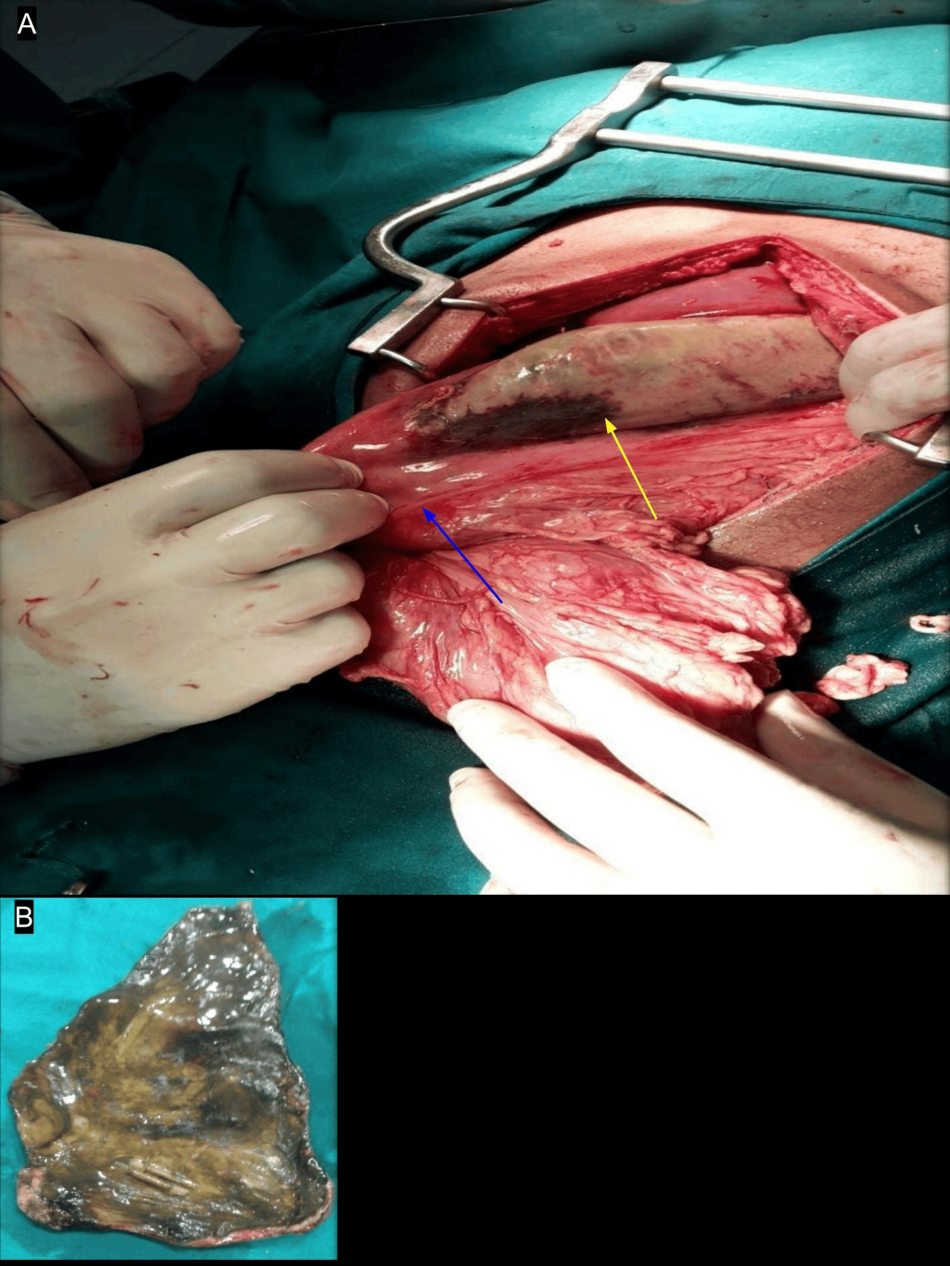
Surgery details Gangrenous patch (A) over the anterior wall of the stomach of size 14 cm (longitudinal) × 8 cm (transverse) involving the fundus, body, and antrum (yellow arrow). Dilated duodenum up to the second part of the duodenum (blue arrow). Greater curvatures were spared. Resected gangrenous patch (B).

**Figure 4:**
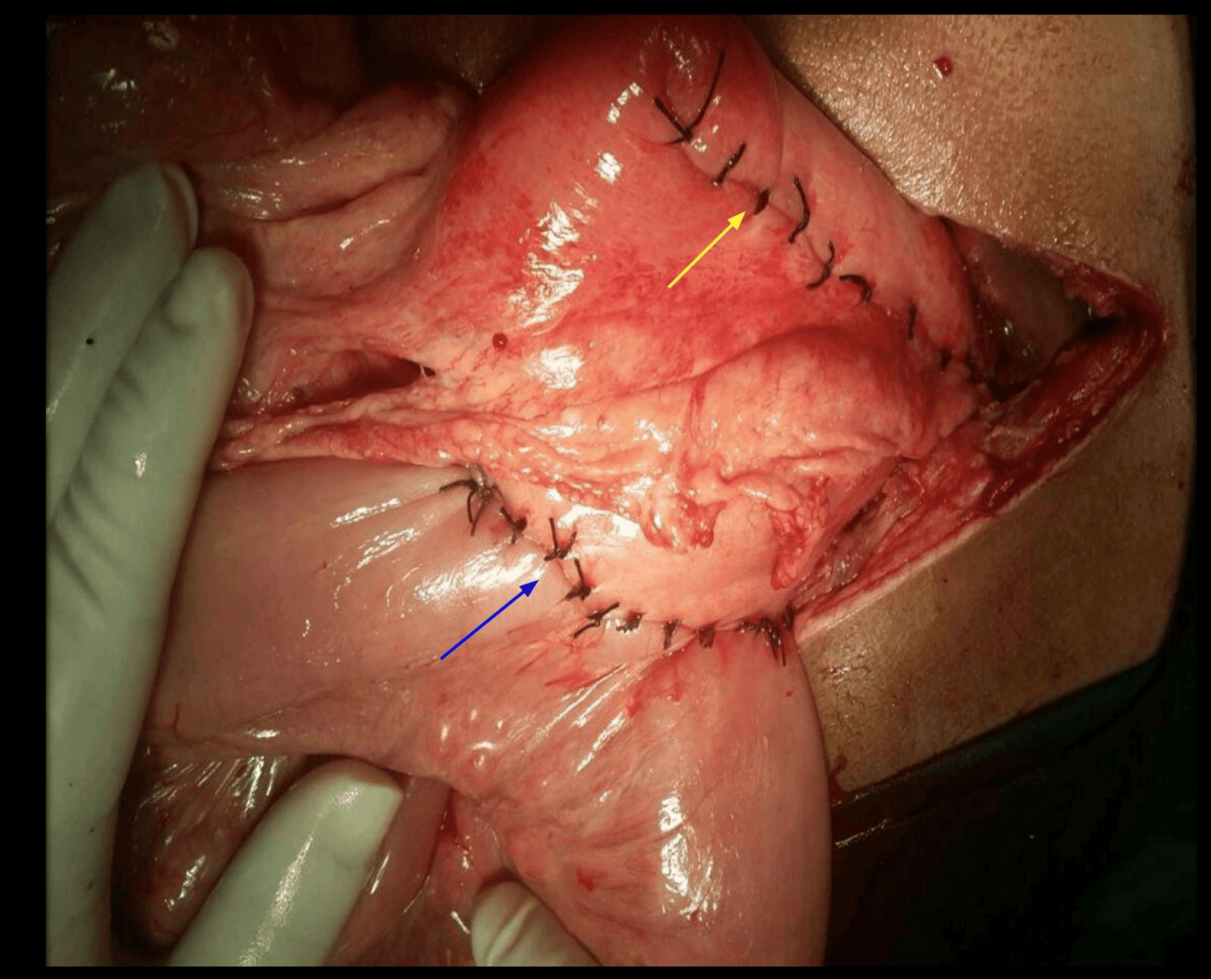
Resection of necrotic patch (yellow arrow) with reconstruction and isoperistaltic retrocolic gastrojejunostomy (blue arrow)

Emergency surgery involved resection of the necrotic gastric segment (Figure 3) and reconstruction via isoperistaltic retrocolic gastrojejunostomy (Figure 4). Intraoperatively, a 14 × 8 cm gangrenous patch was identified on the anterior gastric wall, involving the fundus, body, and antrum, with sparing of both curvatures. The duodenum was dilated up to the second part, with collapsed distal bowel. No evidence of superior mesenteric artery syndrome was found, and the remaining peritoneal cavity and diaphragm were unremarkable.

**Figure 5:**
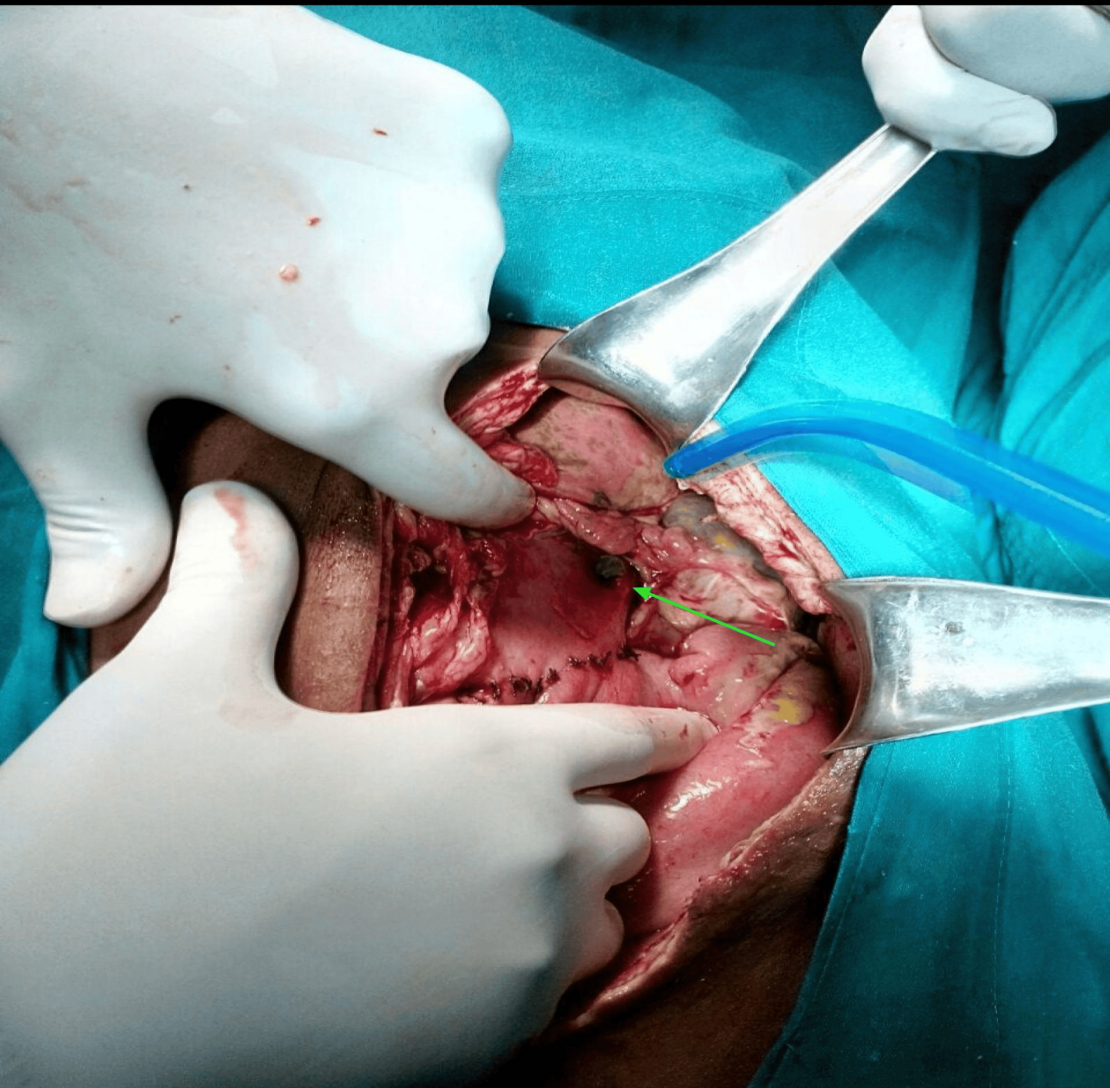
A fresh gastric perforation (green arrow) over the anterior wall near the greater curvature of size 2 × 2 cm was identified during re-exploration on the sixth postoperative day

The postoperative course was initially uneventful until the fifth postoperative day, when the patient developed copious biliary discharge from the surgical wound. Re-exploration revealed a fresh 2 × 2 cm gastric perforation on the anterior wall near the greater curvature, while the gastrojejunostomy anastomosis was intact (Figure 5). The perforation was repaired using a modified Graham’s omental patch, and a nasojejunal tube was placed for early enteral feeding.

Recovery following re-exploration was satisfactory. Enteral feeding resumed on postoperative day three, and a gastrografin dye study on day six confirmed a patent duodenum and gastrojejunostomy. The patient was transitioned to an oral diet, which was well tolerated, with no further complications.

## Discussion

Acute idiopathic gastric necrosis remains one of the most challenging and rare gastrointestinal emergencies, with limited literature documenting its clinical progression and outcomes, particularly in pediatric and adolescent populations [1-4]. The condition is marked by a high mortality rate, with studies reporting deaths in 50-80% of cases when diagnosis and intervention are delayed, highlighting the necessity of early identification and prompt surgical management to improve outcomes. Sharma et al. provided foundational insight into gastric ischemia, categorizing etiologies into local vascular causes, systemic hypoperfusion, and mechanical obstruction [1]. Their study noted abdominal pain, gastrointestinal bleeding, and altered mental status as common presentations. Mechanical obstruction can lead to gastric dilatation, increasing intragastric pressure and impeding perfusion, as seen in our case, a mechanism echoed in prior reports. Pediatric cases are exceedingly uncommon, adding significant value to each reported instance. For example, Trindade et al. described a 13-year-old girl with rapid deterioration from benign symptoms to fatal gastric necrosis despite surgical management, underscoring the aggressive nature and systemic complications of this condition [2]. Conversely, Yorke et al. reported favorable outcomes in an 18-year-old following timely diagnosis and intervention [3]. These comparisons reinforce that early, aggressive surgical management can improve outcomes in young patients with otherwise fulminant disease. Sultanoglu and Demirbakan documented a unique case in an 18-year-old male with intellectual disability, where excessive food intake led to gastric dilatation and near-total gastric necrosis [4]. Total gastrectomy with esophagojejunostomy was necessary, demonstrating that extensive surgical intervention can be lifesaving in cases of profound necrosis and highlighting the need for heightened suspicion in vulnerable populations.

The majority of prior reports document older adults with comorbidities as being at the highest risk. However, age, comorbidity burden, and physiological reserve play vital roles in both presentation and prognosis. Tognoni et al. described idiopathic fundus necrosis, illustrating anatomical variability and emphasizing the importance of surgical planning tailored to the necrosis site [5]. Similarly, González García et al. explored the association of gastric necrosis with eating disorders and mechanical obstruction, presenting varied underlying mechanisms [6]. López et al. demonstrated that localized necrosis, though rare, also carries a grave risk, while Komac et al. highlighted the possibility of successful outcomes in geriatric patients with diligent multidisciplinary care [7,8]. Reviewing literature reveals important age-related patterns: pediatric patients typically present with accelerated progression and poor reserves for coping with systemic inflammation, while adolescents and young adults, as illustrated by Yorke et al. and our present case, may benefit from prompt intervention and lower comorbidity rates. Older adults frequently require more extensive surgery and perioperative support due to underlying diseases.

The optimal surgical approach should be individualized. Localized gastric necrosis may be managed with partial resection and reconstruction, as in our patient, enabling preservation of gastric function and effective decompression. When necrosis is widespread, total gastrectomy may be life-saving, despite its significant long-term implications. Unstable patients may require a staged, damage-control approach [1-4]. Postoperative complications, such as anastomotic leaks or gastric perforations, are not uncommon due to poor tissue integrity and ongoing inflammation. Vigilant clinical monitoring and readiness for prompt re-intervention are essential, as shown by our case of perforation managed successfully with a modified Graham's omental patch. Early enteral nutrition plays a crucial role in postoperative recovery. Outcomes in acute gastric necrosis are determined by several factors, including the timelines of diagnosis and intervention, extent of necrosis, patient age, comorbidities, and quality of perioperative care. Early, aggressive management is consistently associated with improved survival. Comprehensive postoperative care, rapid imaging when indicated, and multidisciplinary involvement are vital to optimize prognosis [1-4,8].

Given the infrequency of this diagnosis, multicenter studies and detailed case series are needed to refine diagnostic criteria, establish predictive risk models, and determine optimal surgical strategies and follow-up protocols. Long-term data on functional and nutritional outcomes are also necessary for patient counseling and rehabilitation strategies [1-8]. Although large-scale studies are limited by the rarity of this entity, the current case literature furnishes invaluable insights into pathophysiology, management, and outcomes, shaping future clinical guidelines. Despite substantial mortality and morbidity, favorable results, such as those seen in our patient, underscore that with early recognition, timely surgery, vigilant postoperative monitoring, and a collaborative multidisciplinary approach, even complex cases of acute idiopathic gastric necrosis can achieve survival and good quality of life [1-4,8].

## Conclusions

Acute idiopathic gastric necrosis represents a surgical emergency with mortality rates of 50-80% when diagnosis is delayed. This case of a 14-year-old boy demonstrates that favorable outcomes are achievable through early recognition, prompt surgical intervention, and vigilant postoperative monitoring. The clinical implications are significant: first, clinicians must maintain high suspicion for gastric necrosis in young patients presenting with acute abdomen and proximal intestinal obstruction features; second, immediate surgical management is essential as conservative treatment rarely succeeds; and third, intensive postoperative care with readiness for re-intervention is crucial given the high complication rates.

The successful management despite postoperative gastric perforation emphasizes that a multidisciplinary approach combining experienced surgical teams, intensive care support, and appropriate nutritional rehabilitation can result in positive outcomes even in complex cases. This rare condition requires continued case reporting to advance understanding of optimal management strategies and improve patient outcomes. Enhanced awareness among emergency physicians, gastroenterologists, and surgeons is essential for early recognition and successful treatment of this life-threatening condition.
